# Understanding concepts of generalism and specialism amongst medical students at a research-intensive London medical school

**DOI:** 10.1186/s12909-022-03355-1

**Published:** 2022-04-18

**Authors:** Adam T. Misky, Ronak J. Shah, Chee Yeen Fung, Amir H. Sam, Karim Meeran, Martyn Kingsbury, Victoria Salem

**Affiliations:** 1 Imperial College School of Medicine, Imperial College London, London, England; 2 Centre for Higher Education Research and Scholarship, Imperial College London, London, England

**Keywords:** Specialist, Generalist, Curriculum, Career

## Abstract

**Background:**

Many prominent UK medical organisations have identified a need for more generalist clinicians to address the complex requirements of an aging society. We sought to clarify attitudes towards “Specialists” and “Generalists” amongst medical students and junior doctors at Imperial College School of Medicine.

**Methods:**

A survey exploring medical students’ beliefs was followed up by qualitative analysis of focus groups of medical students and Imperial-graduate foundation year doctors.

**Results:**

First year medical students associated specialists with academia and higher income, and generalists with ease of training and job availability. Senior (Years 5/6) medical students associated specialists even more firmly with broader influence and academic work, whilst generalists were assigned lower prestige but the same workload as specialists. The medical student focus group discussed concepts of Generalism pertaining only to Primary Care. In contrast, the foundation year doctor focus group revealed that Generalism was now seen to include some hospital care, and the perception that generalists sat lower in a knowledge hierarchy had been challenged.

**Conclusion:**

Perceptions that Generalism is associated with lower prestige in the medical profession are already present at the very start of medical school and seem to be reinforced during undergraduate training. In early postgraduate clinical practice, the perceived knowledge and prestige hierarchy lessens. These findings can help inform curriculum redesign and the promotion of Generalism as a rewarding career aspiration.

**Supplementary Information:**

The online version contains supplementary material available at 10.1186/s12909-022-03355-1.

## Background

The central educational remit of UK medical schools is to deliver curricula that prepare future doctors to practice within the NHS. The General Medical Council (GMC)’s *Outcomes for Graduates* series has for many years identified that modern-day societal health needs a call for more generalists. It highlights the requirements of a diverse population, with increasingly complex medical needs, calling for a shift in balance between specialist services and those provided in wider settings [1]. Crucially, such an approach to care is also aligned with what patients themselves want. In 2013, this was crystallised in the Shape of Training Review [2]:

“Patients and the public need more doctors who are capable of providing general care in broad specialties across a range of different settings. This is being driven by a growing number of people with multiple co-morbidities, an ageing population, health inequalities and increasing patient expectations.”

At the vanguard of this type of care provision are General Practitioners (GPs). The Royal College of General Practitioners (RCGP) vision for the 2022 GP states that: “… expert generalist care is needed more now than at any time since the foundation of the NHS – and this requirement will become greater still over the next decade. Only a healthcare professional with highly developed generalist skills is able to apply his or her medical expertise to the growing range of long-term conditions; to incorporate this knowledge into ‘whole-person’ understanding of the patient and their family; to manage risk safely; and to share complex decisions with patients and carers, while adopting an integrated approach to their care” [3].

The Royal College of Physicians (RCP) highlights the importance of the re-introduction of Generalism into medical training and raising the profile of hospital-based generalists: “Medical education and training will develop doctors with the knowledge and skills to manage the current and future demographic of patients. We need a cadre of doctors with the knowledge and expertise necessary to diagnose, manage and coordinate continuing care for the increasing number of patients with multiple and complex conditions. This includes the expertise to manage older patients with frailty and dementia” [4]. A similar call for more generalists, in particular family practice, has been heard in multiple healthcare systems around the world, often hand in hand with an apparent divergence in graduate aspirations away from general practice [5–7].

However, in reality, medical sciences remain largely taught according to a specialist-based, compartmentalised approach that is at odds with modern-day integrative medicine. General Practice in particular, but all strands of Medical Generalism (for example, Acute or General Internal Medicine), are facing recruitment difficulties [8]. The work pressures faced by over-stretched generalists inevitably feeds into a vicious cycle that makes such a career option less favourable to the next generation. In line with this, the complexities of the relative perceived prestige of Generalism and Specialism within the medical profession should be considered [9]. In a recent collaborative task force, under the joint sponsorship of Health Education England (HEE) and the Medical Schools Council (MSC), the hidden values and expectations firmly entrenched within the medical profession were explored [10]. Many are embedded in the traditional perception of primary care as distinct from, and of lower status than, secondary care; a concept established at the inception of the NHS.

In all UK medical schools, the amount of curriculum time dedicated to community-based learning has increased and yet this is not always commensurate with an increased number of graduates aspiring to or entering generalist careers [11, 12]. Thus, whilst some studies conclude that a critical factor in this choice is exposure to generalist careers at an undergraduate level, we would argue that allocating more time to primary care is not enough and that the quality of this experience as well as access to relatable role models is just as important [7, 13, 14]. The challenge persists of how to deliver core curriculum coverage and impart an appreciation of the value of generalists and the increasing need for expert generalist care. Researchers have identified several factors that influence medical undergraduates’ and postgraduates’ choice to pursue a generalist career, including lifestyle factors, aspects of patient care, career progression, job opportunities and prestige [5–7, 13, 15].

The aim of this study was to capture perceptions of “Specialists” and “Generalists” amongst medical students at Imperial College School of Medicine, a research-intensive medical school in London. A better understanding of these perceptions and possible biases will inform curriculum redesign and the promotion of Generalism in a more progressive way.

## Methods

This study was undertaken with medical students attending Imperial College London (ICL) School of Medicine and Foundation Year 1 doctors (pre-registration, first year medical graduates of ICL). Imperial College London is a research-intensive, STEMM-focussed university with close links to hospitals under the umbrella of Imperial College Healthcare NHS Trust and the associated Academic Health Sciences Centre. ICL distinguishes its medical education programme as having a particularly strong scientific emphasis, noting that the Faculty of Medicine “…has an international reputation for excellence…” and the School of Medicine “harnesses that excellence to provide a unique, research-led student experience in Medicine and Health Sciences.”

### Survey

An anonymous survey was performed in March 2016. Students in Years 1 (junior medical students), 5 and 6 only (senior) were approached to limit survey fatigue and avoid clashes with exam periods in other year groups. This questionnaire (Appendix 1) was developed by two of the investigators (MK and VS) on reflection of personal experience (job roles in Medical Education, Clinical Academia and General Medicine) and a knowledge of the literature surrounding associations with certain career choices amongst medical students [5–7, 13, 15, 16]. The questionnaire was delivered via email using the anonymised Qualtrics platform and prompted respondents to complete slider ratings of eighteen job attributes according to whether they applied more to medical generalists or specialists.

The reason for the scale was to provide the students with an easy way to visually gauge and respond to whether an attribute was weighted more towards their concepts of a “Specialist” or a “Generalist”. An unscaled line was used to mitigate the tendency for responses to cluster around mark points and obtain more individually authentic data. The line was presented on the screen with an arrow starting in the middle. Students had to move the arrow towards the left if they felt that attribute was more associated with Specialism and to the right if it was associated with Generalism. The computer programme then converted this as a measurement from 0–10 (with a 0.1 degree of accuracy). Thus, a score of 5.0 on this scale was classed as that attribute being equally associated with generalists and specialists, a score of 0.0 wholly towards specialists and so on. The term ‘Generalist’ was located on the right of any horizontal scale bar in an attempt to remove any bias in the question layout.

### Focus groups

To explore some of the themes inferred from the survey results in 2016, a Focus Group of six medical students was convened who were in Years 3–4. Two participants were male and four female. In 2020, a second Focus Group was convened consisting of five junior doctors at the beginning of their Foundation Years. All had graduated from ICL, although they were not the same participants as the 2016 group. Two participants were female and three male. The second Focus Group explored the same themes as the first one, with a view to understanding the evolution of attitudes. The focus groups were moderated by an experienced investigator (VS), starting with an opening introduction to discuss notions of Generalism and Specialism (as applied to a career in Medicine), supported by a short list of guiding questions derived from the survey and ultimately more probing questions dependent on some of the responses. This is summarised in the Focus Group guide provided in Appendix 2. In line with guidance [17], questions were designed to be open ended, neutral and attempted to avoid leading language. The entire discussion was audio recorded, transcribed verbatim, anonymised, and thematically analysed.

The project received institutional ethical approval from Imperial College London Medical Education Ethics Committee.

None of the students (surveyed or in the Focus Group) had a direct supervisory relationship with any of the investigators. Involvement in the study was voluntary. An incentive of a £50 prize draw entry was included at the end of the survey invitation. Students who were interested in contributing further were invited to email the investigators to volunteer for a Focus Group. The junior doctors were recruited by word of mouth and via the Education Centre at the Trust, specifically calling for Imperial graduates now working at Foundation Year level. There was no direct educational supervisory relationship with any of the study team. An information sheet was emailed prior to the Focus Groups and informed consent was sought to transcribe and publish anonymised comments. Refreshments were provided but no other incentives. Participants were able to withdraw from the study (including the use of their quotes) up until the point of transcription and anonymisation i.e. up to three months after the Focus Group. No participants withdrew.

### Data analysis

For each survey response, the cursor position on the sliding rating scale for each of the eighteen attributes was converted to a numerical value with 0 representing ‘Specialist’, 5 representing equal and 10 representing ‘Generalist’. The answers were rounded to one decimal place. These numerical values were averaged for each attribute and the attributes subsequently ranked by average numerical value. Assuming a median score in the range of 4 to 6 out of 10 as assigning neutrality, a one-sample Wilcoxon test was used to ascertain which attributes were significantly rated above 6 (i.e. weighted towards generalists) and which attributes were significantly rated below 4 (i.e. weighted towards specialists).

A graphical representation of the difference in average numerical value for the various attributes between junior and senior students was subsequently constructed. For ease of visualisation, the previous average numerical values between 0 and 10 are translated into percentage deviations from the neutral score of 5 (which would mean that an attribute is considered to be equally applicable to both generalists and specialists) and presented as mean ± SEM. Group differences between junior and senior mean percentage deviations from neutral were compared using a Mann–Whitney U test [18]. Graphical representations and statistical analyses were created in GraphPad Prism V8.

VS and RS transcribed and anonymised the Focus Group transcripts. Thematic analysis was performed using a pragmatist grounded theory approach. VS, MK, AM and RS performed an iterative process of identifying themes, at first very concrete and representative of the data, but which became more conceptual with repetitive rounds of discussion and coding [19, 20]. The themes emerged and progressed, were categorised and clustered, with supporting quotes organised as presented in the Results section. This process was performed manually without the help of qualitative software. Authors were not blinded to the year group of the Focus Group participants.

## Results

### Survey of medical student attitudes to concepts of specialism versus generalism

Of the 1024 invited participants, 101 completed questionnaires were received resulting in an overall response rate of 9.9%. Junior students represented 24.7% of responses with an average age of 18.7, 56% identified as female, 44% as male. Senior students comprised the remaining 75.3% of respondents with an average age of 23.8, and a female to male ratio of 53% to 47%. At that time the female to male ratio of our full cohort of junior students was 49% to 51% and for senior students the female to male mix was 55% male and 45% female.

Table 1 reveals how the eighteen attributes were scored (from 0 applying exclusively to specialists through to 10 applying exclusively to generalists). The attributes of doing research (actual median 2.5, *p* =  < 0.001), high income (median score 2.6, *p* < 0.001) and being an academic (median score 2.7, *p* < 0.001) were significantly below the neutral score range of 4–6 and therefore more associated with being a specialist. Conversely, ease of training (median score 6.5, *p* = 0.005) was found to be significantly more associated with being a generalist, with a tendency also to assign greater job availability in generalist roles.

**Table 1:**
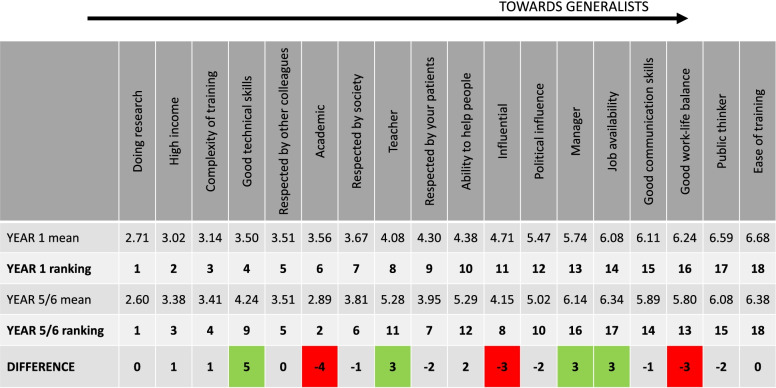
Mean scores for each attribute and their ranking, in ascending order, are represented for Year 1 (*n* = 25) and Year 5/6 (*n* = 76) medical students. Attributes which have moved by three places or more in ranking between Year 1 and Years 5/6 have been highlighted, with red marking attributes that have moved in favour of specialists and green for generalists

**Fig. 1:**
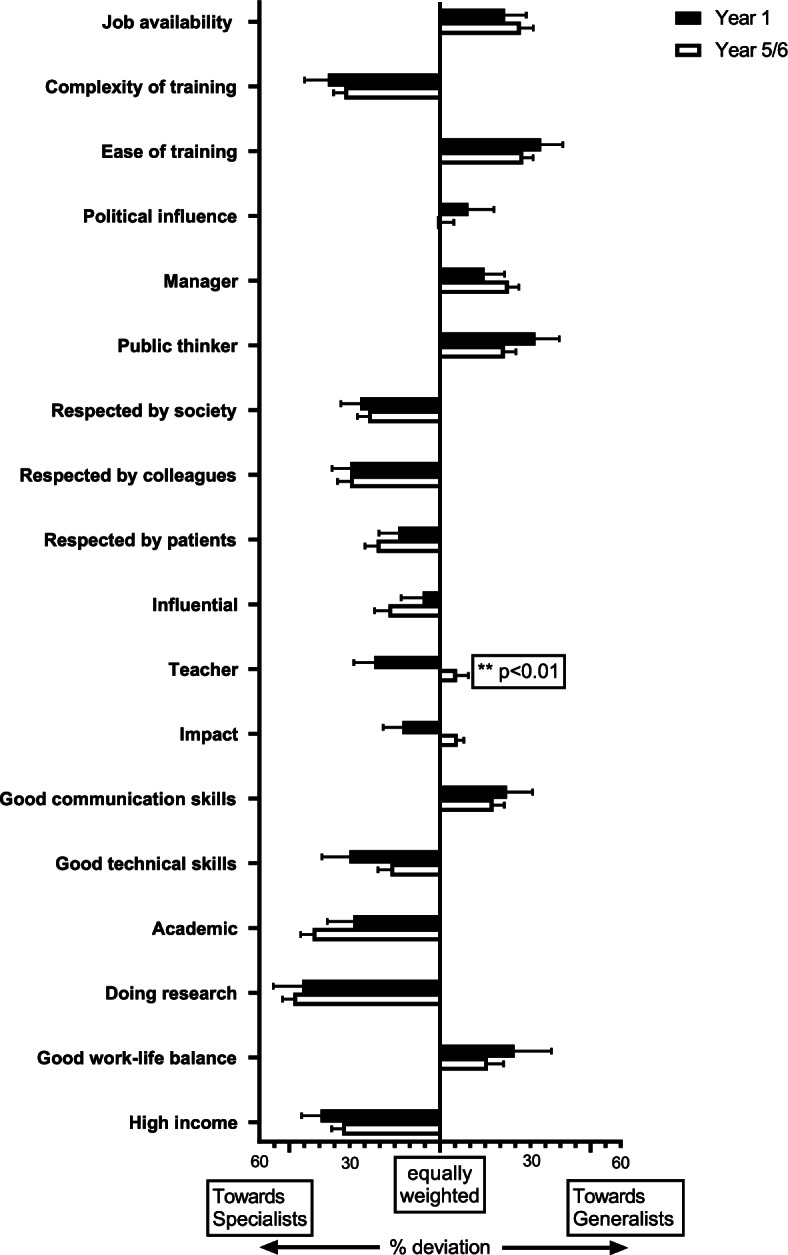
Students were asked to score eighteen professional attributes on a sliding scale, ranging from "applying to specialists" on the far left and "applying to generalists" on the far right. The graph demonstrates percentage deviations from the ‘equally weighted’ score of 5 (which would mean that an attribute is considered to be equally applicable to both generalists and specialists), presented as means ± SEM scores for 101 Imperial College London School of Medicine students in Years 1 and 5/6 of study. Group differences between Year 1 and Years 5/6 mean percentage deviations from neutral were compared using a Mann–Whitney U test

Shifting attitudes to these professional attributes over the Medical School trajectory are examined in Fig. 1. Some views of specialists were confirmed or increased e.g., high income, doing research, being an academic, exerting influence, holding respect (amongst patients, colleagues & society) and training complexity. These attributes seem to be firmly associated with specialists and are either unchanged or become more entrenched over four/five years at Medical School. Work/life balance is not particularly highly rated for any type of doctor, however, an earlier perception that the role of a generalist might be afforded a better work/life balance has begun to be eroded by the end of Medical School – not only do generalists score lower in prestige markers, as scored by senior medical students, but they come to be perceived as having a similar workload as specialists. Concepts of political influence also became more neutral, which may reflect decreased naivete or more realistic views in the older students. Some views of generalists were confirmed or increased e.g., better communication skills, being a manager, ease of training and job availability. Crucially, there was a swing away from specialists towards generalists in some areas. Generalists seem to gain greater standing as teachers and are also relatively ascribed greater technical skills and impact on patients by senior medical students, but this does not affect perceived levels of respect or influence.

### Themes about specialists and generalists from focus group of years 3/4 medical students

The following themes were identified on qualitative analysis of the Focus Group transcript for the Year 3/4 medical students: *Geography of Practice*, *Prestige (including how this relates to Competition), Job Satisfaction, Knowledge, Research, Time (particularly length of training)* and *Teaching/Instruction*. Representative (for the Focus Group) quotes for each of these are given in Table 2.

**Table 2:**
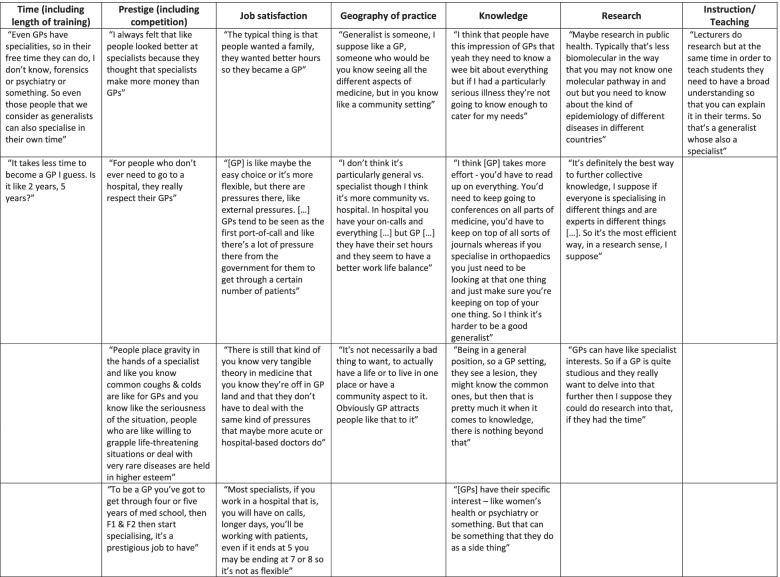
Exemplar quotes for each of the themes defined on theory-driven thematic qualitative analysis of the transcript of a Focus Group of Year 3/4 Imperial College London medical students on the subject of Generalism versus Specialism

In this Focus Group, the discussion surrounding medical professionals who were generalists was limited to GPs, with a consensus that generalists see “all the different aspects of medicine, but in a community setting”. When discussing the overlap between generalists and specialists, the students commented that GPs can have special interests. Interestingly, there is some suggestion that these special interests were linked to areas that are less scientific, more community-based and arguably in the less prestigious sub-discipline areas. Some students noted the generalist nature of certain doctors in a hospital setting (examples given were Endocrinologists and Paediatricians). General Internal Medicine (GIM) was not mentioned.

Generalism was attributed lower prestige, a notion that the students thought was also perceived by members of the public as well as within the profession. This was linked to the belief that generalists earn less money, deal with less medically “serious” situations and that generalist roles are an “easier option to get into”. One commented that GPs are respected by “people who don’t ever need to go to a hospital.” Allied to this prestige hierarchy, is one of knowledge. A ‘breadth versus depth’ dichotomy emerged, with one student stating generalists hold “surface level knowledge” whereas specialists “really delve into it”. Generalists were described as gatekeepers with “nothing beyond common knowledge” and not knowing “enough to cater for my needs” if “I had a particularly serious illness”. A linked concept was that it was not possible to do medical research as a generalist, due to generalists being “less scientific”. None of the students identified this link between scientific pursuit and Specialism as a particular trait of their research-intensive Medical School.

There was an impression that, overall, generalists had better job satisfaction and work/life balance as GPs had “better hours”, were “more flexible” and didn’t have to “deal with the same kind of pressures that maybe more acute or hospital-based doctors do”. One student did acknowledge “external pressures” GPs faced, for example “for them to get through a certain number of patients”. Finally, concepts emerged around the theme of time with the idea that generalists were able to engage in specialist interests “in their own time” or conduct research “if they had the time”. It was also agreed that it takes less time to complete generalist training, perceived by one student as the “easy way out”. It was generally accepted that teaching others (particularly medical students) was a role performed by all doctors, including generalists, and it was acknowledged that being a good teacher required some educational expertise in its own right.

### Evolution of themes about specialists and generalists with the focus group of junior doctors

The transcript of the second Focus Group, undertaken in a group of pre-registration doctors four years after the original Focus Group, was subjected to the same thematic analysis. The themes identified were then stratified according to whether they were an evolution of previous themes or newly emerging ones (Table 3).

**Table 3:**
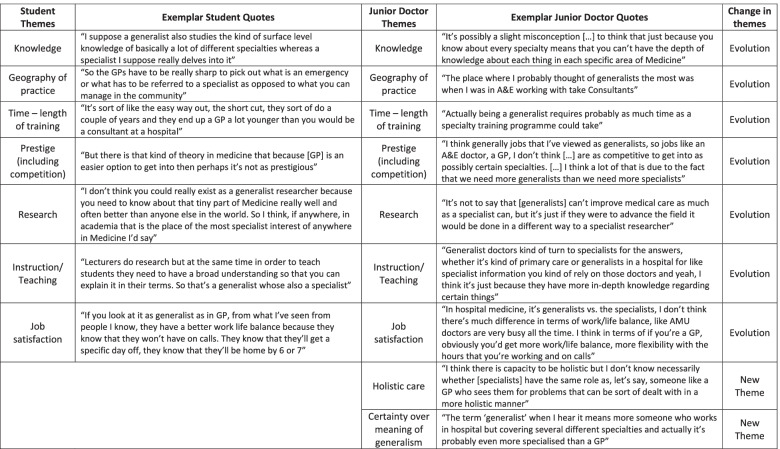
We list the themes that were elicited from the first Focus Group on Specialism versus Generalism in Year 3/4 medical students, with exemplar quotes (columns 1–2). We demonstrate how these themes have evolved amongst junior doctors four years later (columns 3–4) as well as introducing new themes that were not expressed in the medical student Focus Group

Overall, there seemed to be a moderation of the views of Generalism with less extreme characterisation in each of the identified themes. The theme of *Geography* evolved from the idea of generalists providing community and “gatekeeping” services only, to their vital role in hospital medicine as well. This came largely from exposure to the acute medical take and the understanding of how Geriatricians link closely with community practice. The theme of *Prestige* had evolved with the notion that generalist careers may not be as competitive as some specialist career options simply because the health system has more need for generalists. However, there was still a consensus view of Specialism as more prestigious. Nevertheless, the theme of *Knowledge* had evolved from the simplistic idea that generalists have breadth, but no depth of knowledge, to an appreciation that generalist knowledge, while not necessarily the same form, has the same value and complexity. The doctors understood that generalists were more synthetic in their knowledge, able to draw on different strands of experience. Linked to this, the theme of *Time* evolved from a misconception that generalist training is shorter than specialist training, to a new appreciation of how exposure and experience were equally important to the development of an effective and competent generalist.

Related to the new appreciation of the complexity of generalist knowledge came the understanding amongst junior doctors that generalists can engage in *Research*, although this was related to research in hospital management or patient care. The theme of *Instruction/Teaching* had also evolved, applied now not only to medical student teaching but an understanding of how both general physicians and specialists often instruct other doctors by providing guidance on conditions within their field.

In the cohort of junior doctors that engaged with the Focus Group, two new themes were identified. The theme of *Holistic care* emerged with an understanding that while specialists usually look after clinical problems within their field only, generalists often provide more comprehensive care to patients, and this was viewed very positively. As such, there was greater *Certainty about Generalism,* with the junior doctors expressing greater respect for the complexity of the role and understanding that generalists span both community and hospital settings with the same degree of expertise, albeit less easy to crystallise, as specialists.

## Discussion

This study explored the evolution of London medical student ideas about Generalism and Specialism as applied to a career in Medicine. Our data suggest that from early on in the undergraduate course, medical students already have clear biases. Some of these changes are moderated with time, but many key associations become even more established. The culture that is fomented in medical schools is well recognised as a vital shaping force for these perceptions [21]. Both first year and senior medical students associated Generalism with ease of training, greater job availability and more management responsibility whilst Specialism was firmly associated with greater prestige, earning, knowledge and research. Other studies have found similar results through literature review [22] as well as surveying and interviewing medical students about specific specialities, rather than Generalism and Specialism more broadly [23–25]. Creed et al. (2010) found surgery was deemed prestigious and this could be linked to its competitiveness in the authors’ setting [23]. A previous study has also shown how medical students who were surgically-minded rated “reward-related factors” highly in choosing careers, including prestige and pay, suggesting this finding is further nuanced [16]. Compared to junior medical students, senior students were more likely to associate Specialism with scientific rigour, whilst perceptions of a better work/life balance amongst generalists had been eroded. This again chimes with Norredam & Album’s (2007) literature review highlighting “hospital medicine is considered more intellectually demanding than […] general or administrative medicine” [22].

The findings presented here from the questionnaire and Focus Groups tell us that students have little understanding of *both* generalist and specialist career paths, but that there is a perception of both which favours Specialism (in that it is associated with prestige). Related to this, is a particular difficulty associating attributes with generalists, as the students aren’t certain what these roles entail. This perceived prestige imbalance is at odds with what the health service is calling for in the modern era [3, 4]. Some of this bias moderates with experience, but key indicators do not, at least by the time of junior doctor training. This results in the professional and societal need for more generalists being at odds with a skewed perceived prestige economy in medical schools. The question becomes how do we address this, and therefore encourage the brightest and best into Generalism, where they are needed?

Building on this, we engaged a group of Year 3/4 medical students to discuss these themes in more detail. At this mid-way point in their undergraduate medical education, these students revealed a tenuous understanding of the roles and skills of a generalist which was closely linked to notions of the prestige hierarchies that exist within Medicine. These ideas were already present in first year students, and it is unclear if they were already present when they arrived or quickly inculcated after enrolment. This has been shown in other work too that focused on medical students’ impression of speciality prestige [23]. Rather than breaking down these prejudices, the students’ medical education experience bolstered many of them, and generalists strikingly were viewed as having less knowledge in return for less competitive career routes. Strikingly, the concept of a ‘pay-off’ with a greater quality of life for generalists was also diminished, possibly with exposure to the actual pressures of community-based roles, although in this study we did not collect data on perceived attractiveness of certain career options.

These concepts about generalists seemed to have evolved in a meaningful way by the end of the first year of practice post-graduation. Given the references made to experiences on the job it is possible that this transformation was driven by exposure in the workplace. All Focus Group attendees had undertaken at least one rotation in Acute Medicine, but had variously also been exposed to General Surgery, General Practice and Psychiatry as Foundation Year 1 doctors. The key transition we found was an appreciation of the depth of knowledge and synthetic ability to balance numerous factors in clinical decision making required of an outstanding generalist. This requires a more nuanced appreciation of the skills required of a medical practitioner than a simply summative description of specialist facts. Interestingly, the alignment of Specialism with academic rigour also loosened with this transition.

As medical educators, we have an obligation to rebuild narratives about prestige hierarchies in Medicine. This is important from the perspective of justice, but also to bolster the drive and satisfaction of doctors entering generalist career paths, and thereby help meet the changing societal needs as described by the GMC’s *Outcomes for Graduates* [1]. Although medical curricula have evolved greatly to approach systems more holistically and to expose students to General Practice much earlier, we present evidence that this is not enough to alter misconceptions about Generalism or to provide much insight into the role of hospital-based general physicians and surgeons.

The Shape of Training review announced the key message that “Patients and the public need more doctors who are capable of providing general care in broad specialties across a range of different settings.” The lack of understanding of the practice of Generalism has been previously identified and explored by both individual researchers as well as national providers of medical education [8, 10]. Medical students have been shown to have an understanding of Generalism, but not of the practice of Generalism and an entrenched view that primary care in particular is of lower status than secondary care [8, 10]. There is research exploring medical students’ attitudes to General Practice, however there is a distinct scarcity of research exploring medical students’ attitudes to and understanding of hospital-based generalists. Medical schools need to be able to convey a balanced view of generalist career paths and popularise generalist skills and careers amongst medical students. Whilst wholesale re-branding is clearly untenable, our research suggests that one way of approaching this is to highlight the specialist skillsets employed by generalists (that take just as long to master) and are going to be increasingly needed by an aging population presenting with complex co-morbidities. In a research-intensive medical school such as ICL, the entrenched preconceptions about generalists as exempt from undertaking research might be an obvious place to start. We did not compare the attitudes of our students with those from other institutions with different mission statements. However, given the misconception and the need, research-intensive medical schools should not just provide a balanced view but promote and develop the Specialism of General Medicine and more proactively and explicitly step-up research in the Specialism of Generalism. Alternatively, teaching sessions that mirror the essence of being a generalist – that is, managing patients with unselected presenting complaints and incorporating a wide range of factors in the clinical decision-making process – may be a more realistic way of exposing undergraduates to the complexities and rewards of Generalism. Senior doctors also have a role to play in minding the language that is used to describe both their own specialty and others.

Although research on this area is limited, there is some evidence to suggest that the most impactful factors in popularising hospital Generalism are a pre-existing interest in general medicine at the time of enrolment and undertaking an elective in general medicine at the end of medical school [26]. Indeed, Health Education England (HEE) have advised an increased provision of work experience placements at general practice prior to enrolment in medical school in order to increase recruitment to general practice [10]. There is evidence to show that attending general practice work experience placements prior to medical school increases the appeal of that particular generalist career [27]. There is further research to suggest that the amount of time spent in a specialty at medical school does not predict the popularity of that specialty as a career path [28]. We must then consider new ways of sparking undergraduate interest in generalist careers and realigning prestige hierarchies in Medicine.

To date, most of the focus in the UK has been on enhancing generalist experience and training at the postgraduate level. The latest iteration of this is HEE’s trailblazer pilot of the ‘School of Generalism’. The ‘School of Generalism’ embeds a generalist development programme within foundation training for newly qualified doctors, which places graduates in a range of cross disciplinary posts. As integrated generalist development programmes become more commonplace in postgraduate training, medical schools must also adapt their curricula to support the development of more generalist doctors [29].

However, as we have shown, many career choices and biases exist from earlier on in the student career journey, and it remains imperative that medical schools also deal with this issue. Medical schools should incorporate formal modules or domains throughout the undergraduate programme with explicit generalist learning outcomes [30]. Problem-based learning (PBL) may be a practical approach to synthesising clinical knowledge obtained in specialist rotations with generalist skills [31]. The use of PBL in undergraduate teaching has been found to increase interest in general medicine, primary care and holistic clinical practice [32]. In our medical school, generalist skills have been purposefully woven throughout the Clinical Pharmacology and Prescribing domain which crosses all speciality rotations and all year groups. The teaching uses PBL alongside a patient-centred approach to holistically manage realistic clinical scenarios, involving generalist topics such as multimorbidity, population health and sustainability.

Mentorship and role modelling also play a key role in encouraging student career choices [16]. Meaningful exposure to generalist role models will likely increase uptake in generalist careers. However, with most hospital consultants currently trained as specialists, there remains a gap for mentors and role models until more generalist consultants qualify. Medical schools may also want to consider creating more research and quality improvement opportunities for students within Generalism. This may deepen students’ clinical understanding of the field and as well as raising its perceived prestige as an academically vigorous discipline [33].

This study reveals that preconceptions about Generalism exist early on and many negative concepts are further enhanced by medical school. Positive examples of expert generalists can offset these concepts in the early postgraduate years, but for many this may well be too late. This issue can't be solved by a single medical school. It is clearly a challenge for the profession and society to help adjust the perceived prestige of Generalism so that it is more appropriately aligned with society's present and future needs.

### Limitations

It is important to appraise our conclusions and consider some limitations of the study. Firstly, it was conducted in a single centre and therefore we cannot automatically generalise our findings to other UK or international institutions. However, many of the themes raised here have been reported globally, as discussed in our opening section. This study was designed as an interpretive case study to go beyond describing local context, but to use an in-depth knowledge of that context to explore ideas about Specialism and Generalism. It is not designed to be representative in a positivistic way but rather to provide a situated authenticity with wider relevance to explore these complex ideas.

We purposely intended to avoid burdening students with surveys and to avoid exam periods so only three of the six years of students were sent our questionnaire, and no reminders were sent. This explains the slightly low response rate, although the demographic split of respondents was highly representative. The risk of missing some student views was mitigated by ensuring the youngest and oldest medical students were approached to take part. Therefore, we believe we were still able to capture important data about how transition through medical school shapes medical student views on Generalism and Specialism.

We chose a Focus Group format rather than individual structured interviews in order to enhance the texture of the conversation by encouraging interactions and debate between the students/junior doctors themselves rather than depending entirely on the interaction between the investigator and individual participants. Whilst the investigator was trained in interview techniques it remains of course important to recognise that her own personality and experiences, as both a practicing acute medic and clinical academic, will have informed the questioning and interpretation of results.

## Supplementary Information

**Additional file 1. Appendix 1: **Year Group Survey. **Appendix 2: **Focus Group Guide for the study: Understanding Concepts of Generalism and Specialism amongst Medical Students at a Research-Intensive London Medical School.

## Data Availability

The datasets generated and/or analysed during the current study are not publicly available as they contain potentially sensitive and identifiable information but are available from the corresponding author on reasonable request.

## Abbreviations

GIM: General internal medicine
GMC: General Medical Council
HEE: Health Education England
ICL: Imperial College London
MSC: Medical Schools Council
PBL: Problem-based learning
RCGP: Royal College of General Practitioners
RCP: Royal College of Physicians
