# Supplementary material for: Understanding concepts of generalism and specialism amongst medical students at a research-intensive London medical school
Source: BMC Med Educ. 2022 Apr 18;22:291. doi: 10.1186/s12909-022-03355-1 (PMC9017034; doi:10.1186/s12909-022-03355-1)
Supplement: Supplementary file 1 — Additional file 1. Appendix 1: Year Group Survey. Appendix 2: Focus Group Guide for the study: Understanding Concepts of Generalism and Specialism amongst Medical Students at a Research-Intensive London Medical School. [file 12909_2022_3355_MOESM1_ESM.docx]

**Understanding Concepts of Generalism and Specialism amongst Medical Students at a Research-Intensive London Medical School**

**APPENDICES**

**Appendix 1:** Year Group Survey

This was sent out to Year 1, Year 5 and Year 6. Responses from Year 1 were analysed separately to the combined responses of Years 5/6.

**Question 1 (free text response)**

What is your understanding of the term “Generalist” in terms of a career in Medicine?

**Question 2 (free text response)**

What is your understanding of the term “Specialist” in terms of a career in Medicine?

**Question 3 (a rating bar will appear for each of the terms listed below)**

Please move the arrow to rate whether you think the following terms apply more towards a generalist or a specialist doctor:

Specialist Generalist

**Move arrow**

1. High income
2. Good work-life balance
3. Doing research
4. Academic
5. Good technical skills
6. Good communication skills
7. Ability to impact upon/help people
8. Teacher
9. Influential
10. Respected by your patients
11. Respected by other colleagues
12. Respected by society
13. Public thinker
14. Manager
15. Political influence
16. Ease of training
17. Complexity of training
18. Job availability at the end of training

And finally, what is your gender? M/F

What is your age? …………years

**Appendix 2**

Focus Group Guide for the study: **Understanding Concepts of Generalism and Specialism amongst Medical Students at a Research-Intensive London Medical School**

| Question Type | Aim | Purpose | Examples |
| --- | --- | --- | --- |
| UMBRELLA | Opening question about the content of the research question, in a non-leading manner. | **To initiate the discussion and encourage participants to relate their views.** | *I’d like to start by asking each of you to share your concepts of specialising versus generalising, that is, a career in medicine as a specialist or a generalist.* |
| CORE QUESTIONS | Questions that relate directly to the research question – these were derived from the data obtained in our anonymised surveys. | **Answer the research question by aiding participants to discuss in an open, exploratory way. To incorporate the mirroring of responses to ensure that the participants are in agreement.** | *So you brought out the concept there of a General Practitioner, and generalism having a community feel. Any other viewpoints?*  *But which of those 2 job descriptions, generalist versus specialist, do you think is associated with a better work life balance or quality of life and why?*  *So what about ease of training? Is it easier to become a specialist or a generalist?*  *How so you think that the average person on the street who doesn’t really understand medical careers perceives specialists versus generalists?* |
| PLANNED FOLLOW UP QUESTIONS | Specific questions that ask for more details about particular aspects of the core questions | **These questions are asked dependent on participant responses and are planned to answer specific aspects or gain greater detail about initial responses.** | *Any other specialties that you can think of that are associated with being a general physician?*  *You suggested that if a generalist wants to do research they might have to do some of it in their spare time, what does that mean exactly?*  *,*  *What exactly do you mean by breadth rather than depth?*  *You said that the further you are from the hospital, the more likely you are to be a generalist, do you want to expand on that?* |
| UNPLANNED FOLLOW UP QUESTIONS | Questions that arise during the interview based on participant responses | **To gain deeper insight or “unpack” responses.** | *There is generalism from the sense of it being holistic and requiring quite a broad understanding of all of the body. Could you expand on that?*  *You seemed to suggest that it has something to do with lack of seniority and that a generalist is something that you start off as before you become a specialist?*  *And do you think that specialists are less prone to being doubted? You suggested that GPs are more accessible and therefore more likely to be questioned?*  *You said that now you are IN “the system”, your concept of prestige is based on job competitiveness. What was it based on before?*  *You said that a generalist is somebody who’s got a lot of breadth of knowledge but not a lot of depth of knowledge. Do you think there might be a value judgement in that statement?* |
